# Editorial: Sphingolipids in Infection Control

**DOI:** 10.3389/fcell.2021.697290

**Published:** 2021-06-21

**Authors:** Jürgen Seibel, Sibylle Schneider-Schaulies, Burkhard Kleuser

**Affiliations:** ^1^Department for Organic Chemistry and Insitute for Virology and Immunobiology, Julius Maximilian University of Würzburg, Würzburg, Germany; ^2^Institute of Pharmacy, Free University of Berlin, Berlin, Germany

**Keywords:** sphingolipid, infection, regulation, membrane, microdomain

## Introduction

In spite of the availability of preemptive strategies, infectious diseases continue to be a major threat worldwide. Therefore, there is an urgent demand for continuous development of anti-infective and immune-therapeutic strategies in particular for conditions where conventional interventive means are not available, prohibited, or fail. It was particularly in the more recent past, sphingolipids (SLs) have been explored as crucial molecules in infection processes and targeting sphingolipid (SL) metabolism seems to be a novel strategy for intervention in infectious diseases.

As they are major components of cellular membranes, accumulation levels and turnover of SL species effectively take part in cellular processes involving membrane integrity and dynamics (Hannun and Obeid, 2008; Airola and Hannun, 2013). Biosynthesis and metabolism of SLs are highly complex and—except for initiation of their biosynthesis and irreversible degradation via hydrolysis by sphingosine-1-phosphate lyase—accumulation of any SL species is highly dynamic because they rapidly interconvert due to the activity of a plethora of metabolizing enzymes. Adding further complexity to this system, SL species substantially vary with regard to acyl chain length, saturation, hydroxylation or complexity of their head groups, and compartmentalization of their complex metabolism as exerted by a multitude of enzyme isoforms involved in these processes (excellently reviewed in Hannun and Obeid, 2008; Gault et al., 2010; Feng et al., 2018; Harayama and Riezman, 2018).

Because of their high abundance, the composition of SLs within membranes strongly impacts on their biophysical properties by regulating deformability and fluidity (as important in inward/outward vesiculation), compartmentalization of membrane proteins and associated membrane proximal signaling molecules and hence, signal initiation and cytoskeletal dynamics (Bollinger et al., 2005; Grassme et al., 2007; Hannun and Obeid, 2008). In addition, bioactive SL species such as ceramides, ceramide-1-phosphate, sphingosine and sphingosine-1-phosphate actively take part in signaling processes regulating key cellular processes including cell survival, activation and apoptosis (Schenck et al., 2007; Dimanche-Boitrel et al., 2011; Takabe and Spiegel, 2014; Spiegel et al., 2019). Seminal studies on the role of SL dynamics in various disease pathologies established therapeutic concepts and development of drugs targeting their turnover which has been particularly successful in treatment of sphingolipidoses, atherosclerosis, cystic fibrosis and cancer (Kolter, 2011; Ryland et al., 2011; Schulze and Sandhoff, 2011). In view of progress made in SL regulation at a cellular basis, development of experimental tools also including highly specific inhibitors and genetic ablation strategies as well as experimental test systems, modulation of SL metabolism as therapeutic approach will predictably increase (Loewith et al., 2019).

Membrane dynamics, receptor sorting, cytoskeletal rearrangements and vesicular trafficking are also centrally involved in infection processes by controlling both the interaction of a pathogen with its host cell or trafficking and activity of immune (effector) cells. This Research Topic aimed at providing insight into progress made in this still emerging field in SL research.

## Research Contribution of the Collection to the Field

### Advances in Technology and Their Implementation

Mechanistical studies there have long been hampered by the lack of suitable tools and test systems which are being continuously developed. The highly cited study of Wigger et al. describes a novel methodology that for the first time allows to monitor the entire ER-associated SL *de novo* biosynthesis involving stable-isotope labeling and liquid chromatography-mass spectrometry.

The microsome based assay does allow for both modulations of this pathway *in vitro*, but also to monitor its alterations in cells or tissues also including immune cells. In addition to isotope labeled, functionalized precursors have been successfully used more recently for this purpose (Fink et al., 2021). Implementation of bio-orthogonal chemistry to generate functionalized compounds has also greatly advanced and enabled detection and trafficking of SLs in fixed and living cells (Haberkant and Holthuis, 2014; Kuerschner and Thiele, 2014). In a primary astrocyte model system, cross-linkable functionalized ceramide analogs and proximity ligation assays were combined to visualize proteins associated with ceramide-enriched platforms (Jiang et al.). Applied in this paper to ceramide tubulin and VDAC1 ceramide complexes, this technique will substantially advance studies on co-detection of proteins in ceramide-enriched microdomains and regulation of this particular interactions. SLs have also been successfully resolved by super-resolution microscopy before (Burgert et al., 2017) and this technique, combined with structured illumination microscopy (SIM) has now been used to unravel accumulation and coating of meningococci with GM1 ganglioside upon uptake into HBMEC (Schlegel et al.).

This study allowed to reveal the importance of cell cycle dependent GM1 increase at the plasma membrane for the efficiency of uptake rather than binding of the pathogen. It thereby unifies substantial advances in a technical approach to visualize SL dynamics and support for their essential role in pathogen host interactions during bacterial infection.

### The Role of SL Dynamics in Infection at the Level of Pathogens

Three comprehensive reviews focus on the modulatory capacity of SLs within the life cycle of several pathogenic bacteria ranging from attachment and uptake, formation of intracellular compartments, and interaction with cell autonomous defense mechanisms. Most interestingly, Rolando and Buchrieser report on bacterial strategies, especially bacterially encoded enzymes mimicking SL metabolizing enzymes. These enable them to actively regulate the catabolic SL pathway in eukaryotic cells for their individual demands to counteract host responses and facilitate intracellular growth, and are therefore promising targets for intervention. Kunz and Kozjak-Pavlovic also elaborate on the role of SLs in attachment and uptake of bacteria, however, in common to Banhart et al., focus on sphingolipid mediated regulation of cellular signaling pathways promoting intracellular trafficking and survival. Amongst those, special emphasis is given to formation of a cellular inclusion compartment. This is essential for survival and reproduction of *Chlamydia trachomatis*, and relies for *C. trachomatis* and other chlamydia and chlamydia-like micro-organisms, on acquisition of sphingomyelin and ceramide from the host cell by as yet ill-defined transport mechanism. As referred to in all three reviews, particular SL species proved to be important for the successful interaction of bacteria with their hosts and therefore, host enzymes or bacterial gene products promoting their production might be targets for therapeutic intervention. Less well-investigated to date, targeting of SL metabolizing enzymes can also act antivirally. An example for this is provided by Grafen et al. who report sensitivity of measles virus (MV) replication in lymphocytes to inhibition of the acid ceramidase and sphingosine kinase, most reflecting the inhibitor-induced reduction of cellular metabolic activity (Grafen et al.).

Noteworthy, certain SL species including sphinganine, sphingosine and ceramides, can also directly act as antimicrobials and thereby possibly represent a novel class of antibiotics, as detailed by Kunz and Kozjak-Pavlovic.

As most recently revealed, this may also apply to viral infections (Lang et al., 2020).

The therapeutic potential of another SL subclass, glycosphingolipids (GSL), is discussed by Aerts et al. who review the dual role of those in pathogen binding and uptake, and in regulating immune cell functions important in infection control. Given that they therefore might regulate both processes, therapeutics targeting GSL biosynthesis as already approved for treatment of lysosomal glycolipid storage diseases, might, as discussed by the authors, represent new avenues for infection control.

### SL Dynamics Involved in Immune Control of Pathogens

Six contributions elaborate on the impact of SL dynamics in immune cells thereby controlling infections at a cellular rather than the pathogen level. As established, sphingosine-1-phosphate (S1P) gradients effectively promote recruitment of immune cells, also including macrophages. This particular SL emerged, however, as potent regulator of macrophage function and thereby, as of crucial pathogenic importance in infectious and non-infectious diseases as comprehensively reviewed by Weigert et al.. In fact, S1P has proven to be relevant in protection against *Mycobacterium tuberculosis* via its impact on macrophage differentiation (Nadella et al.).

Several contributions to this Research Topic focus on the role of sphingolipid homeostasis in T cells and regulation thereof in response to pathogen challenge.

Hollmann and colleagues highlight current knowledge on the impact of ceramide generation on activation, differentiation and effector functions in distinct T cell populations (Hollmann et al.).

Reporting on studies involving newly generated tools such as mouse strains deficient for or overexpressing sphingolipid-metabolizing enzymes, they discuss recent progress made with regard to translational approaches aiming on T cell modulation in suitable animal (infection) models as a basis for further development in clinical use in humans. Notably, drugs already in clinical use for treatment of non-infectious diseases are amongst the potential candidates for differential modulation of T cell subpopulations. Direct experimental proof for a beneficial effect of regulating the SL pool at the level of sphingomyelin breakdown in T cells is provided by Hose et al. There, T cell specific overexpression of the acid sphingomyelinase (ASM) was protective in *Plasmodium yoelii* infected mice by enhancement of T cell mediated immunity. In line with ASM activity being supportive to T cell immunity, ASM ablation was found to augment susceptibility to *C. rodentium* infection in mice used as a model of mucosal immunity (Meiners et al.).

Strikingly, in this model ASM deficiency was associated with uncontrolled inflammatory T_h_1 and T_h_17 responses and thereby, progressive colonic pathology. Supporting that ablation of ASM might indeed differentially affect T cell subpopulations, the proportion of regulatory T cells among CD4^+^ T cells was found elevated in ASM deficient mice, and this was associated with progressive CNS infection in a mouse model earlier (Hollmann et al., 2016).

Finally, two contributions focus on the specific role of sphingomyelin breakdown in T cells and its role in regulating T cell activation at a cellular level. Using Jurkat T cells deficient for the neutral sphingomyelinase 2 (NSM2), Börtlein et al. clearly revealed that this enzyme is of importance for the plasma membrane (PM) composition under homeostatic conditions. Its absence most prominently affected transport of PM cholesterol to the endoplasmic reticulum and production of cholesteryl esters (CE) there. Importantly, prevention of CE production (upon NSM2 ablation or inhibition of cholesterol acetyltransferases) significantly impaired T cell receptor (TCR) driven expansion of both, CD4+ and CD8+ T cells, indicating that the NSM2 activity is of importance for T cell expansion. Within their comprehensive review, Avota et al. provide insight into the role of ASM and NSM2 (and other SL metabolizing enzymes) in various aspects of T cell activation mainly including T cell viability, relay of signals given by TCR or upon co-stimulation or T cell motility and tissue homing. Not surprisingly, the activity of SL metabolizing enzymes needs stringent spatiotemporal control also in T cells where their deregulation by MV results in impaired T cell activation.

## Concluding Remarks

In summary, understanding the role of SL dynamics in infection control can still be regarded as emerging field of research that bears substantial promise for highly fascinating insights into pathogen-host interactions and modulation of immunity, and thereby, development of therapeutics. With the substantial progress made in techniques and tools to study, quantify, visualize and specifically target and modulate SL metabolism as well as the availability of (multi) functionalized SL species in the recent past, achievement of this ambitious goal and transfer into clinical application is very likely to become possible.

## Author Contributions

JS, SS-S, and BK wrote and corrected the manuscript. All authors contributed to the article and approved the submitted version.

## Conflict of Interest

The authors declare that the research was conducted in the absence of any commercial or financial relationships that could be construed as a potential conflict of interest.
